# HDAC-inhibition counteracts everolimus resistance in renal cell carcinoma in vitro by diminishing cdk2 and cyclin A

**DOI:** 10.1186/1476-4598-13-152

**Published:** 2014-06-16

**Authors:** Eva Juengel, Snigdha Nowaz, Jasmina Makarevi, Iyad Natsheh, Isabella Werner, Karen Nelson, Michael Reiter, Igor Tsaur, Jens Mani, Sebastian Harder, Georg Bartsch, Axel Haferkamp, Roman A Blaheta

**Affiliations:** 1 Department of Urology, Goethe-University, Interdisciplinary Science Building. Building 25A, Room 404, Theodor-Stern-Kai 7, Frankfurt / Main D-60590, Germany; 2 Royal Scientific Society, Amman, Jordan; 3 Department of Thoracic and Cardiovascular Surgery, Goethe-University, Frankfurt am Main, Germany; 4 Department of Vascular and Endovascular Surgery, Goethe-University, Frankfurt am Main, Germany; 5 Department of Clinical Pharmacology, Goethe-University, Frankfurt am Main, Germany

**Keywords:** Renal cell carcinoma, Everolimus resistance, HDAC-inhibition, cdk2/cyclin A, Tumor growth

## Abstract

**Background:**

Targeted therapies have improved therapeutic options of treating renal cell carcinoma (RCC). However, drug response is temporary due to resistance development.

**Methods:**

Functional and molecular changes in RCC Caki-1 cells, after acquired resistance to the mammalian target of rapamycin (mTOR)-inhibitor everolimus (Caki^res^), were investigated with and without additional application of the histone deacetylase (HDAC)-inhibitor valproic acid (VPA). Cell growth was evaluated by MTT assay, cell cycle progression and apoptosis by flow cytometry. Target molecules of everolimus and VPA, apoptotic and cell cycle regulating proteins were investigated by western blotting. siRNA blockade was performed to evaluate the functional relevance of the proteins.

**Results:**

Everolimus resistance was accompanied by significant increases in the percentage of G2/M-phase cells and in the IC_50_. Akt and p70S6K, targets of everolimus, were activated in Caki^res^ compared to drug sensitive cells. The most prominent change in Caki^res^ cells was an increase in the cell cycle activating proteins cdk2 and cyclin A. Knock-down of cdk2 and cyclin A caused significant growth inhibition in the Caki^res^ cells. The HDAC-inhibitor, VPA, counteracted everolimus resistance in Caki^res^, evidenced by a significant decrease in tumor growth and cdk2/cyclin A.

**Conclusion:**

It is concluded that non-response to everolimus is characterized by increased cdk2/cyclin A, driving RCC cells into the G2/M-phase. VPA hinders everolimus non-response by diminishing cdk2/cyclin A. Therefore, treatment with HDAC-inhibitors might be an option for patients with advanced renal cell carcinoma and acquired everolimus resistance.

## Background

Over the past years the therapy for renal cell carcinoma (RCC) has undergone change, with better understanding of the molecular biology of RCC leading to the development of several targeted agents. The phosphatidyl-inositol-3 kinase (PI3K)/Akt/mammalian target of rapamycin (mTOR) pathway has been identified as a pivotal key regulator. mTOR has an impact on various cellular functions, including cell growth, proliferation and cell survival [1]. Two mTOR-inhibitors, temsirolimus (Torisel®) and everolimus (Afinitor®), have been approved by the FDA to treat advanced RCC due to prolonged progression free survival [2-4]. However, targeted therapy is not curative in metastatic RCC and drug response is limited. Recently, it has been shown that chronic mTOR-inhibition evokes undesired feedback mechanisms in RCC cells, which may lead to resistance development [5,6]. Undesirable feedback has also been demonstrated in prostate cancer cells after chronic exposure to everolimus, indicating molecular alterations tied to acquired resistance [7]. Agents targeting such feedback loops and cross talk with other pathways involved in acquired resistance to mTOR-inhibition are, therefore, urgently required [8]. Studies and clinical trials have demonstrated that histone deacetylase (HDAC)-inhibitors in combination with other therapies, including targeted therapies, induce synergistic or additive anti-tumor effects [9-11]. It has also been reported that during chronic application of everolimus, combination with the HDAC-inhibitor valproic acid (VPA) contributes to sustained anti-tumor activity [6]. Additionally, HDAC-inhibitors have been shown to re-sensitize tumor cells to cytotoxic drug treatment [12,13]. Hence, HDAC-inhibition might prove promising in reversing everolimus resistance in RCC. To follow up on a pilot study employing everolimus resistant RCC Caki-1 cells (Caki^res^) [6], resistance dependent functional and molecular aberrations were investigated in the same cell line. Further investigation was designed to determine whether Caki^res^ cell growth could be influenced by the HDAC-inhibitor VPA, whereby the growth behavior of Caki^res^ compared to VPA treated Caki^res^ cells was evaluated. It is shown that everolimus resistance contributes to a significant increase in the IC_50_, an elevated percentage of G2/M-phase cells and distinct up-regulation of the cell cycle activating proteins cdk2 and cyclin A. VPA counteracted everolimus resistance by significantly inhibiting tumor growth and reducing cdk2 and cyclin A. Thus, VPA might represent a new promising treatment option for RCC patients with acquired everolimus resistance.

## Results

### Exposure to everolimus induced resistance in RCC cells

**Figure 1:**
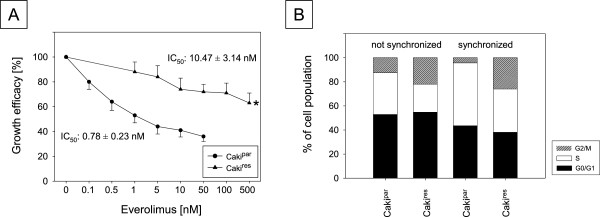
**Dose response analysis of Caki**^**par **^**and Caki**^**res**^**. (A)** Growth efficacy of increasing concentrations of everolimus on Caki^par^ and Caki^res^ cell growth from 24 h to 72 h. *Indicates significant difference to controls, set to 100% (SD ≤ 16%, n = 6). IC_50_-values of Caki^par^ and Caki^res^ cells were calculated from dose response analysis. **(B)** Cell cycle analysis of unsynchronized and synchronized Caki^par^ and Caki^res^ cells in subconfluent cell cultures. The cell phase is expressed as percentage of total cells. The figure shows one of five separate experiments.

24 h exposure to ascending concentrations of everolimus [0.1-1 μM] induced a dose dependent significant reduction in the number of Caki^par^ cells compared to the untreated control (set to 100%, Figure 1A) with an IC_50_ of 0.78 ± 0.23 nM. Everolimus resistance (Caki^res^) was evidenced by a significant shift of the IC_50_ to 10.47 ± 3.14 nM.

### Resistance towards everolimus significantly enhanced the G2/M-phase

Evaluation of cell cycle progression revealed significant alterations after acquired everolimus resistance. The G2/M-phase percentage was increased in unsynchronized Caki^res^ cells, compared to Caki^par^, and was accompanied by a decrease in the S-phase (Figure 1B). Synchronization of the cells led to a similar shift, additionally reducing the percentage of G0/G1-phase cells in Caki^res^ (Figure 1B).

### Re-treatment of Caki^res^ with therapeutic everolimus concentrations caused an increase in the G2/M-phase

**Figure 2:**
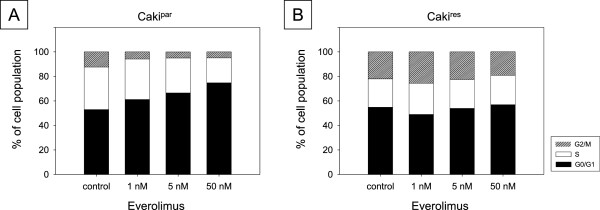
**Cell cycle analysis of Caki**^**par **^**(A) and Caki**^**res **^**(B) after 24 h exposure to 1, 5 and 50 nM everolimus in subconfluent cell cultures.** The cell population is expressed as percentage of the total cells analyzed. Untreated cells served as controls. One representative experiment of six is shown.

Treatment of Caki^par^ for 24 h with 1, 5 or 50 nM everolimus dose-dependently reduced S- and G2/M-phase cells, while the percentage of G0/G1-phase cells increased (Figure 2A). Re-treatment with everolimus had no significant effect on any cell phase in Caki^res^, regardless of the concentration (Figure 2B). Therefore, all further re-treatment investigation was performed with 1 nM everolimus.

### Resistance-dependent alteration in tumor growth was associated with modulated protein expression

**Figure 3:**
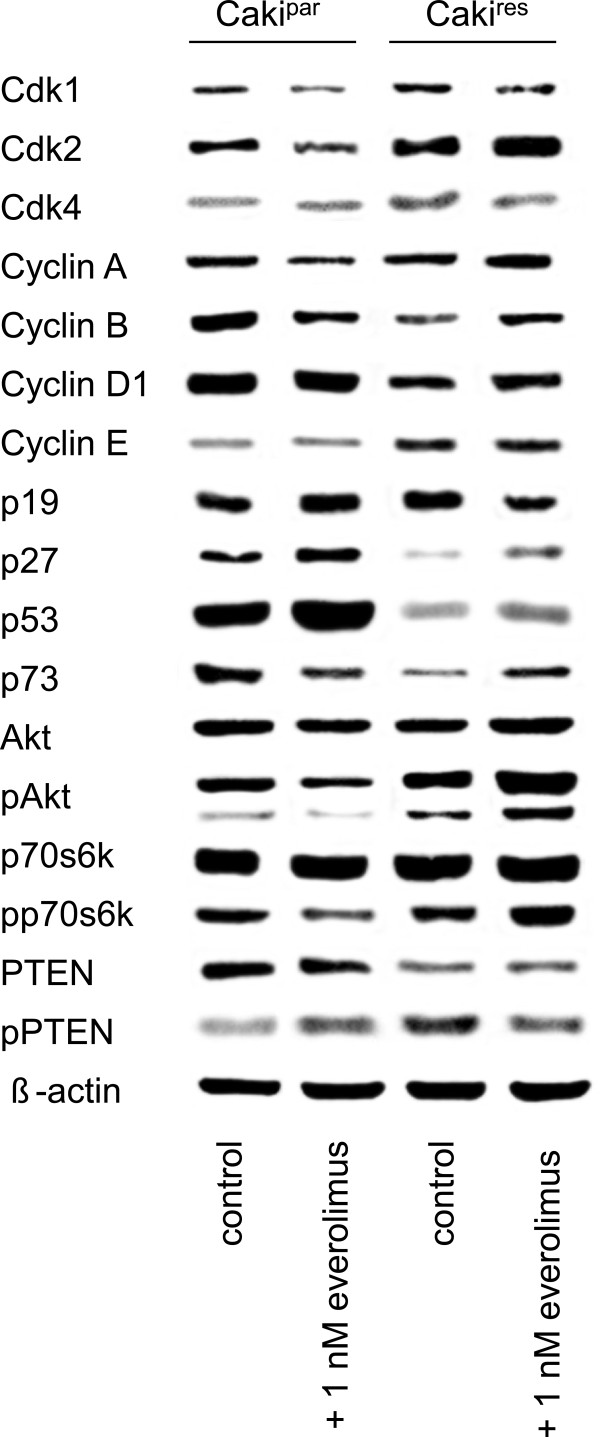
**Western blot analysis of cell cycle regulating proteins and everolimus target molecules.** Caki^par^ and Caki^res^ treated with 1 nM everolimus for 24 h. Controls remained untreated. β-actin served as the internal control. The figure shows one representative from three separate experiments.

After 24 h exposure to 1 nM everolimus, Caki^par^ revealed a decrease in phosphorylated Akt (pAkt) and p70S6 kinase (pp70S6K) compared to untreated Caki^par^ (Figure 3). Concomitantly, Akt’s negative regulator PTEN (pPTEN) was activated by 1 nM everolimus. The 24 h application of 1 nM everolimus to Caki^par^ caused a distinct decrease in the cell cycle activating proteins cdk1 and cdk2 as well as in cyclin A and cyclin B, whereas the negative cell cycle regulator p27 was elevated. Compared to Caki^par^, Caki^res^ displayed an activation of pAkt and considerable elevation of cdk1, cdk2, cdk4 and cyclin E, whereas p27, p53 and p73 were diminished. Re-treating Caki^res^ with 1 nM everolimus evoked additional activation of pAkt and pp70S6K, a further augmentation of cdk2 and cyclin A, along with deactivation of pPTEN. However, the expression of p27, p53 and p73 was elevated in Caki^res^ after re-treatment.

### The HDAC-inhibitor VPA inhibited tumor growth in Caki^par^ and Caki^res^

**Figure 4:**
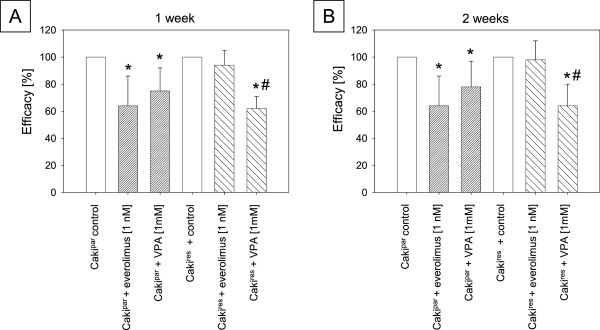
**Cell growth analysis.** Growth inhibitory efficacy of 1 nM or 1 mM VPA in Caki^par^ and Caki^res^ cells treated for one **(A)** or two weeks **(B)**. *Indicates significant difference to untreated controls, set at 100%. ^#^Indicates significant difference between Caki^par^ + VPA and Caki^res^ + VPA (SD ≤ 22%, n = 6).

Application of the HDAC-inhibitor VPA [1 mM] to Caki^par^ cells for 1 or 2 weeks contributed to a significant reduction in cell growth (Figure 4A,B), although to a lesser extent than that from 1 nM everolimus exposure. Exposing Caki^res^ to VPA [1 mM] also led to significantly diminished tumor growth (Figure 4A,B). The VPA induced growth inhibition in Caki^res^ was significantly higher than that in Caki^par^.

### Resistance-dependent alterations in protein expression were counteracted by HDAC-inhibition through VPA

**Figure 5:**
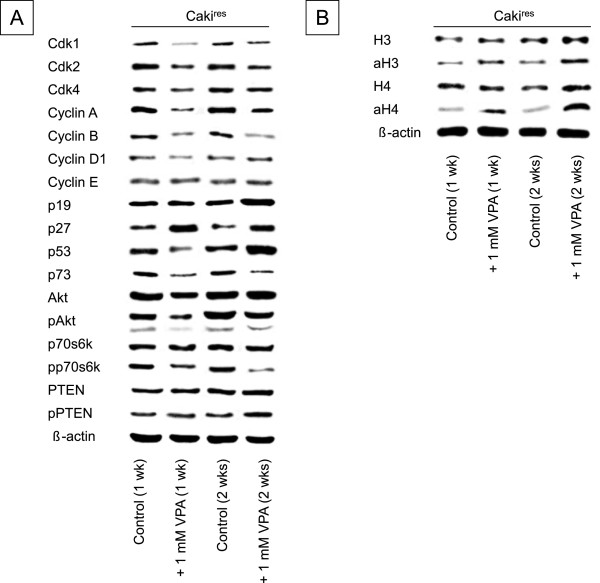
**Western blot analysis of Caki**^**res **^**exposed to 1 mM VPA for one (1 wk) or two weeks (2 wks) showing cell cycle regulating proteins and target molecules of mTOR- (A) and HDAC-inhibitors (B).** VPA untreated cells served as controls. β-actin served as the internal control. The figure shows one representative from three separate experiments.

VPA caused deactivation of pAkt and pp70S6k in Caki^res^ after 1 or 2 weeks (Figure 5A). In addition, the expression of Akt and p70S6k was diminished with VPA after 1 week. In contrast, the activity of pPTEN was enhanced after 1 or 2 weeks of VPA treatment, compared to untreated Caki^res^. Applying VPA for 1 or 2 weeks to Caki^res^ caused a considerable decrease in cdk2 and cyclin A and an elevation in p27 (Figure 5A). VPA treatment resulted in increased acetylation (1 or 2 weeks) and increased total content of histone H3 and H4 (2 weeks) in Caki^res^ (Figure 5B).

### Resistance towards everolimus did not affect apoptosis in RCC

Apoptosis was not influenced by treatment with everolimus in either Caki^par^ or Caki^res^ (data not shown). In good accordance, examination of the apoptosis proteins caspase 3 and PARP by western blot showed no differences between Caki^par^ and Caki^res^ and no changes were apparent after treatment with VPA (data not shown).

### siRNA knock-down

**Figure 6:**
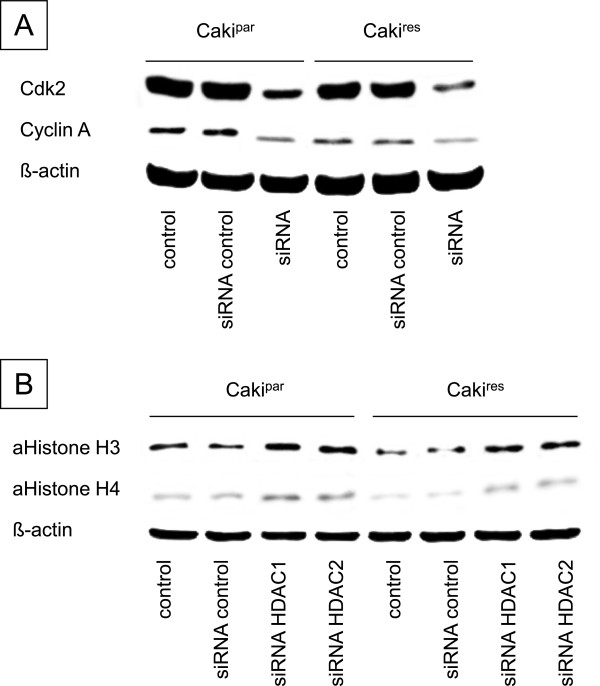
**siRNA knock-down controls.** siRNA blockade of cdk2 or cyclin A **(A)** and HDAC1 or HDAC2 **(B)** were confirmed by western blotting. Caki^par^ and Caki^res^ cells were transfected with cdk2, cyclin A, HDAC1 or HDAC2 siRNA. Untreated cells and with control siRNA transfected cells served as controls. One representative from three experiments is shown.

**Figure 7:**
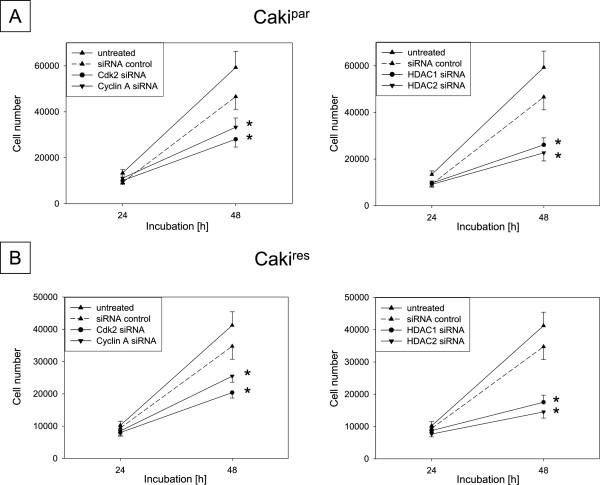
**Influence of cdk2, cyclin A, HDAC1 or HDAC2 knock-down on tumor cell growth.** Caki^par^**(A)** and Caki^res^**(B)** cells were transfected with cdk2, cyclin A HDAC1 or HDAC2 siRNA and then subjected to the MTT cell growth assay. Untreated cells and with control siRNA transfected cells served as controls. Cell number of control Caki^par^ or Caki^res^ cells and the corresponding knock-down cells were measured. *Indicates significant difference to untreated and siRNA controls (SD ≤ 18%, n = 6).

Since cdk2 and cyclin A were distinctly increased in RCC^res^ and were mainly affected by VPA treatment, their functional relevance during resistance dependent tumor growth was evaluated by siRNA knock-down. Cdk2 and cyclin A siRNA blockade, verified by western blot analysis (Figure 6A), resulted in significant growth inhibition in both Caki^par^ and Caki^res^, compared to untreated and siRNA controls (Figure 7A + B left). The impact of HDAC1 and HDAC2 as targets of VPA was also determined by siRNA blockade. HDAC1 and HDAC2 siRNA knock-down contributed to an increase in histone H3 and H4 acetylation in Caki^par^ and Caki^res^ (Figure 6B). The observed elevation of histone H3 and H4 acetylation was accompanied by significantly reduced tumor growth in Caki^par^ and Caki^res^, compared to untreated and siRNA controls (Figure 7A + B right).

## Discussion

Chronic everolimus treatment led to drug resistant RCC cells. It was possible to hinder resistance by applying the HDAC-inhibitor VPA.

Caki^res^ revealed a 13-fold higher IC_50_ than Caki^par^. This IC_50_ change is within the range of the 4 to 22 fold-change used to define drug-resistance [14,15], indicating clear-cut everolimus resistance. The IC_50_ shift was associated with a considerable increase in the G2/M-phase, whereby S-phase cells were shifted into the G2/M-phase and the G0/G1-phase fraction was reduced. Such a shift has also been observed during lung cancer drug resistance with an accelerated G2/M-phase transition [16]. In prostate cancer cells everolimus resistance has also revealed a higher G2/M-phase cell cycle fraction [7]. Based on a recent study, chronic everolimus application to RCC cells resulted in an accumulation of G2/M-phase cells [6]. The G2/M-shift may, therefore, be characteristic of chronic everolimus exposure and be associated with resistance development.

Everolimus resistance contributed to characteristic molecular changes, including activation of the everolimus target molecules Akt and p70S6K. Re-treatment of Caki^res^ with 1 nM everolimus evoked additional activation of Akt and p70S6k, whereas the activity of the Akt negative regulator, PTEN, was diminished. Akt is a key molecule with multiple functions, including cell growth and survival. Tumor progression and resistance development in RCC in vitro and in vivo towards different agents has been associated with increased activity of the PI3K/Akt/mTOR signaling pathway [5,8,17,18]. Enhanced activity of Akt has also been shown to be involved in bone metastasis, larger tumor size, grades III/IV tumors and shorter disease-free survival in RCC [19-21]. Moreover, elevated Akt phosphorylation has been associated with hyperproliferation and overexpression of cell cycle proteins [22]. Indeed, the present study shows that the cell cycle activating proteins cdk2 and cyclin A were both over-expressed in Caki^res^ compared to Caki^par^, and further increased after re-treatment with everolimus. The finding that proteins involved in mitotic control were further up-regulated after applying a therapeutic everolimus concentration is clinically relevant, since mitotic activity of tumor cells is often accelerated, once resistance has developed. In the present investigation the number of mitotic cells significantly increased when Caki^res^ cells were exposed to low-dosed everolimus. This finding might, therefore, explain why RCC patients, treated with temsirolimus or everolimus often develop progressive disease [23]. The same progression has been observed in different gynecologic cancers as well as estrogen receptor-positive breast cancer and colorectal carcinoma, indicating a correlation between treatment resistance and enhanced aggressiveness characterized by accelerated tumor growth [24-26].

The functional relevance of cdk2 and cyclin A in tumor growth was verified by siRNA knock down, revealing significant growth inhibition after cdk2 and cyclin A loss. Cdk2 and cyclin A establish complexes in the S-phase and are required for entrance into the G2/M-phase. Indeed, low expression of cdk2 and cyclin A has been shown to be associated with cell cycle arrest and accumulation of cells in the S-phase [27]. Everolimus resistance has also been associated with a considerable increase in cdk2 in prostate cancer [7] and in RCC cells [6]. Thus, augmented cdk2 seems closely related to non-responsiveness towards everolimus and deserves attention in overcoming resistance development. High levels of cyclin A have been associated with a worse outcome and have been proposed as a prognostic factor in breast cancer, as well [28-30]. Similarly, a cyclin A increase in RCC has been associated with elevated tumor size and poor survival [31]. In good accordance with the present findings regarding Caki^res^, cyclin A expression has been shown to be inversely correlated with the expression of the cell cycle negative regulator p27 in RCC [31]. It may, therefore, be concluded that resistance development towards everolimus is accompanied by elevated cdk2/cyclin A, driving tumor cells from the S- into the G2/M-phase, leading to a more aggressive tumor phenotype with enhanced growth capacity.

The HDAC-inhibitor VPA caused a significant decrease in RCC tumor growth. Since VPA’s growth inhibitory effect on Caki-1 was even more pronounced in Caki^res^ than in Caki^par^, VPA seems to re-sensitize the tumor cells to everolimus. However, it may also be concluded that chronic treatment with everolimus sensitizes the cells to VPA treatment. Although this is speculative, various studies have shown that HDAC-inhibitors in combination with everolimus induce synergistic anti-tumor effects [10]. HDAC-inhibitors have been implicated in the re-sensitization of tumor cells to cytotoxic drug treatment [12,13] and concomitant application of VPA with chemo- or targeted therapies has shown that VPA prevents tumor cells from becoming resistant [6,32]. VPA may, therefore, counteract resistance dependent feedback loops and reverse molecular alterations in everolimus resistant cells. VPA treatment did deactivate proteins associated with mitosis in the Caki^res^ cells, particularly Akt and p70S6k. Both cdk2 and cyclin A were strongly enhanced in Caki^res^ and were considerably reduced in the presence of VPA. Thus, cdk2 and cyclin A could serve as predictive indicators for a response to VPA. In several tumor entities application of VPA for up to 2 weeks has resulted in counter-regulation of the cdk-cyclin axis, contributing to significant growth inhibition [6,33]. Since cdk2/cyclin A reduction and growth inhibition in Caki^res^ after application with VPA was accompanied by acetylation of histone H3 and H4, epigenetic modification might be involved in the anti-tumor effect. Other investigators have also reported an association between histone H3 and H4 acetylation, cdk2 reduction and diminished growth in bladder and prostate cancer cells [33,34]. Knock-down of HDAC1 and HDAC2, responsible for deacetylation of histone H3 and H4 [35,36], has induced considerable acetylation of histone H3 and H4 in Caki^res^, correlating with significant growth inhibition. The present investigation also indicates that histone H3 and H4 acetylation diminishes tumor growth, probably by influencing the cell cycle activating proteins cdk2/cyclin A.

## Conclusion

The present findings reveal that resistance development towards the mTOR-inhibitor, everolimus, is associated with undesired feedback loops, including activation of the Akt/mTOR signaling pathway and increased cdk2/cyclin A expression, and is associated with cell cycle progression and tumor growth. Evidence is provided that re-treatment with everolimus not only fails to inhibit tumor progression in Caki^res^, but activates progression. Since resistance is a serious problem in treating RCC the HDAC-inhibitor VPA could be employed to impair cdk2/cyclin A expression. Acetylation of histone H3 and H4 plays a pivotal role in this process, significantly inhibiting tumor cell growth. Patients with renal cell carcinoma and acquired everolimus resistance might, therefore, benefit from treatment with VPA. In vivo investigation and clinical trials are necessary to verify tumor growth inhibition by VPA in everolimus resistant renal cell carcinoma.

## Methods

### Cell culture

Kidney carcinoma cells, Caki-1, were purchased from LGC Promochem (Wesel, Germany). The cells were grown and subcultured in RPMI 1640 medium (Seromed, Berlin, Germany) supplemented with 10% FCS, 20 mM Hepes-buffer, 100 IU/ml penicillin and 100 μg/ml streptomycin at 37°C in a humidified, 5% CO_2_ incubator.

### Drugs

Everolimus (RAD001, Afinitor™, Novartis Pharma AG, Basel, Switzerland) was dissolved in DMSO as a 10 mM stock solution and stored as aliquots at -20°C. Prior to experiments, everolimus was diluted in cell culture medium. Resistance towards everolimus was induced by treating Caki-1 cells with stepwise ascending concentrations from 1 nM up to 1 μM. The tumor cells were further exposed to 1 μM everolimus twice a week for over one year. Tumor cells, resistant to everolimus, were designated Caki^res^ and control cells, sensitive to everolimus, were designated Caki^par^.

Besides comparing characteristics of Caki^res^ to Caki^par^, the response to therapeutic everolimus concentrations (drug re-treatment) was also investigated. Preparation for everolimus re-treatment was carried out by incubating Caki^res^ cells for three days with everolimus-free medium. Subsequently, 1, 5 or 50 nM everolimus was applied to the Caki^res^ and Caki^par^ cells.

Valproic acid (VPA) (G. L. Pharma GmbH, Lannach, Austria) was applied at a final concentration of 1 mM to Caki^res^ and Caki^par^ cells twice a week over a total of either 1 or 2 weeks. Control cell cultures remained untreated.

To evaluate toxic effects of applied drugs, cell viability was determined by trypan blue (Gibco/Invitrogen, Darmstadt, Germany).

### Apoptosis

To detect apoptosis the expression of Annexin V/propidium iodide (PI) was evaluated using the Annexin V-FITC Apoptosis Detection kit (BD Pharmingen, Heidelberg, Germany). Tumor cells were washed twice with PBS-buffer, and then incubated with 5 μl of Annexin V-FITC and 5 μl of PI in the dark for 15 min at room temperature. Cells were analyzed on a FACScalibur (BD Biosciences, Heidelberg, Germany). The percentage of vital, necrotic and apoptotic cells (early and late) in each quadrant was calculated using Cell-Quest software (BD Biosciences).

### Measurement of tumor cell growth and proliferation

Cell growth was assessed using the 3-(4,5-dimethylthiazol-2-yl)-2,5-diphenyltetrazolium bromide (MTT) dye reduction assay (Roche Diagnostics, Penzberg, Germany). RCC cells (50 μl, 1×10^5^ cells/ml) were seeded onto 96-well tissue culture plates. After 24, 48 and 72 h MTT (0.5 mg/ml) was added for an additional 4 h. Thereafter, cells were lysed in a buffer containing 10% SDS in 0.01 M HCl. The plates were incubated overnight at 37 °C, 5% CO_2_. Absorbance at 550 nm was determined for each well using a microplate ELISA reader. Each experiment was done in triplicate. After subtracting background absorbance, results were expressed as mean cell number. IC_50_ values were calculated on the basis of the dose response analysis of Caki^par^ and Caki^res^ using GraphPad Prism 5.0.

### Cell cycle analysis

Cell cycle analysis was performed with RCC cultures grown to subconfluency and carried out after 24 h using both asynchronous and synchronous cell populations. Caki-1 cells were synchronized at the G1-S boundary with aphidicolin (1 μg/ml) 24 h before starting cell cycle analysis and subsequently resuspended in fresh (aphidicolin free) medium for 2 h. Asynchronous or synchronous tumor cell populations were stained with propidium iodide using a Cycle TEST PLUS DNA Reagent Kit (Becton Dickinson) and then subjected to flow cytometry with a FACScan flow cytometer (Becton Dickinson). 10,000 events were collected from each sample. Data acquisition was carried out using Cell-Quest software and cell cycle distribution, calculated with ModFit software (Becton Dickinson). The number of gated cells in the G1-, S- or G2/M-phases were expressed in%.

### Western blot analysis

To investigate cell cycle regulating proteins in Caki-1 cells, tumor cell lysates (50 μg) were applied to polyacrylamide gels (7-15%) and electrophoresed for 90 min at 100 V. The protein was then transferred to nitrocellulose membranes. After blocking with non-fat dry milk for 1 h, the membranes were incubated overnight with monoclonal antibodies directed against cell cycle proteins: cdk1 (mouse IgG1, clone 1, dilution 1:2,500), cdk2 (mouse IgG2a, clone 55, dilution 1:2,500), cdk4 (mouse IgG1, clone 97; dilution 1:250), cyclin A (mouse IgG1, clone 25, dilution 1:250), cyclin B (mouse IgG1, clone 18, dilution 1:1,000), cyclin D1 (mouse IgG1, clone G124-326, dilution 1:250), cyclin E (mouse IgG1, clone HE12, dilution 1:1,000), p19 (mouse IgG1, clone 52/p19, 1:5,000), p21 (mouse IgG1, clone 2G12, dilution 1:250), p27 (Kip1, mouse IgG1, clone 57, 1:500), p53 (mouse IgG2b, clone DO-7, dilution 1:500) and p73 (mouse IgG1, clone ER-15, dilution 1:500, all: BD Biosciences). Apoptotic effects, the protein expression of caspase 3 and PARP (both: rabbit IgG, dilution 1:1,000, Cell Signaling Technology by New England Biolabs GmbH, Frankfurt, Germany) were also investigated. To evaluate target specificity of everolimus and VPA, mTOR signaling and histone acetylation were evaluated. The following monoclonal antibodies were employed to determine mTOR signaling: Akt (PKBα/Akt, clone 55), phospho Akt (pAkt; clone 104A282, both: mouse IgG1, dilution 1:500, BD Biosciences), p70S6k (clone 49D7), phospho p70S6k (pp70S6k; clone 108D2), PTEN (phosphatase and tensin homolog) and phospho PTEN (Ser380/Thr382/383, all: rabbit IgG, dilution 1:1,000, Cell Signaling Technology). To investigate histone acetylation, cell lysates were marked with anti-histone H3 (rabbit IgG, clone Y173, dilution 1:5,000), anti-acetylated H3 (rabbit IgG, clone Y28, dilution 1:500, both: Epitomics, USA), anti-histone H4 (rabbit IgG, clone: N/A, dilution 1:250, Imgenex, USA) and anti-acetylated H4 (Lys8, rabbit IgG, dilution 1:500, Upstate Biotechnology, USA). HRP-conjugated goat-anti-mouse or goat-anti-rabbit IgG (both: dilution 1:5,000, Upstate Biotechnology, Lake Placid, NY, USA) were used as secondary antibodies. The membranes were briefly incubated with ECL detection reagent (ECL™, Amersham/GE Healthcare, München, Germany) to visualize the proteins and exposed to an x-ray-film (Hyperfilm™ EC™; Amersham/GE Healthcare). β-actin (dilution 1:1,000, Sigma, Taufenkirchen, Germany) served as the internal control.

### siRNA blockade

Caki-1 cells (3×10^5^/6-well) were transfected with small interfering RNA (siRNA) directed against cdk2 (gene ID: 1017, target sequence: AGGTGGTGGCGCTTAAGAAAA), cyclin A (gene ID: 890, target sequence: GCCAGCTGTCAGGATAATAAA), HDAC1 (gene ID: 3065, target sequence: CACCCGGAGGAAAGTCTGTTA) or HDAC2 (gene ID: 3066, target sequence: TCCCAATGAGTTGCCATATAA; all: Qiagen, Hilden, Germany) with a siRNA/transfection reagent (HiPerFect Transfection Reagent; Qiagen, Hilden, Germany) ratio of 1:6. Untreated cells and cells treated with 5 nM control siRNA (All stars negative control siRNA; Qiagen, Hilden, Germany) served as controls. Knock-down was verified by western blot analysis. Tumor cell growth was analyzed by the MTT assay as indicated above.

### Statistics

All experiments were performed 3-6 times. Statistical significance was investigated by the Wilcoxon-Mann-Whitney-U-test. Differences were considered statistically significant at a p-value less than 0.05.

## Competing interests

The authors declare that they have no competing interests.

## Authors’ contributions

EJ was involved in the study conception, data analysis, statistics and drafted the manuscript. SN performed growth and cell cycle analysis. JaM and IN accomplished the western blot analysis. IW and MR examined apoptotic effects. IT and JeM did siRNA blockade. KN and GB were involved in data interpretation and critically revising the manuscript. SH made the statistics. AH and RAB have designed the study and gave final approval of the manuscript version to be published.

## Authors’ information

Axel Haferkamp and Roman A Blaheta contributed equally as senior authors.
